# HSP90 inhibitors potentiate PGF_2α_-induced IL-6 synthesis via p38 MAP kinase in osteoblasts

**DOI:** 10.1371/journal.pone.0177878

**Published:** 2017-05-19

**Authors:** Kazuhiko Fujita, Haruhiko Tokuda, Gen Kuroyanagi, Naohiro Yamamoto, Shingo Kainuma, Tetsu Kawabata, Go Sakai, Rie Matsushima-Nishiwaki, Osamu Kozawa, Takanobu Otsuka

**Affiliations:** 1 Department of Orthopedic Surgery, Nagoya City University Graduate School of Medical Sciences, Nagoya, Japan; 2 Department of Pharmacology, Gifu University Graduate School of Medicine, Gifu, Japan; 3 Department of Clinical Laboratory/Biobank of Medical Genome Center, National Center for Geriatrics and Gerontology, Obu, Aichi, Japan; Universite de Nantes, FRANCE

## Abstract

Heat shock protein 90 (HSP90) that is ubiquitously expressed in various tissues, is recognized to be a major molecular chaperone. We have previously reported that prostaglandin F_2α_ (PGF_2α_), a potent bone remodeling mediator, stimulates the synthesis of interleukin-6 (IL-6) through p44/p42 mitogen-activated protein (MAP) kinase and p38 MAP kinase in osteoblast-like MC3T3-E1 cells, and that Rho-kinase acts at a point upstream of p38 MAP kinase. In the present study, we investigated the involvement of HSP90 in the PGF_2α_-stimulated IL-6 synthesis and the underlying mechanism in MC3T3-E1 cells. Geldanamycin, an inhibitor of HSP90, significantly amplified both the PGF_2α_-stimulated IL-6 release and the mRNA expression levels. In addition, other HSP90 inhibitors, 17-allylamino-17demethoxy-geldanamycin (17-AAG) and 17-dimethylamino-ethylamino-17-demethoxy-geldanamycin (17-DMAG) and onalespib, enhanced the PGF_2α_-stimulated IL-6 release. Geldanamycin, 17-AAG and onalespib markedly strengthened the PGF_2α_-induced phosphorylation of p38 MAP kinase. Geldanamycin and 17-AAG did not affect the PGF_2α_-induced phosphorylation of p44/p42 MAP kinase and myosin phosphatase targeting subunit (MYPT-1), a substrate of Rho-kinase, and the protein levels of RhoA and Rho-kinase. In addition, HSP90-siRNA enhanced the PGF_2α_-induced phosphorylation of p38 MAP kinase. Furthermore, SB203580, an inhibitor of p38 MAP kinase, significantly suppressed the amplification by geldanamycin, 17-AAG or 17-DMAG of the PGF_2α_-stimulated IL-6 release. Our results strongly suggest that HSP90 negatively regulates the PGF_2α_-stimulated IL-6 synthesis in osteoblasts, and that the effect of HSP90 is exerted through regulating p38 MAP kinase activation.

## Introduction

Heat shock proteins (HSPs) are induced in response to biological stress such as heat stress and chemical stress [1]. HSPs, which are generally recognized as molecular chaperones, facilitate the refolding of nonnative proteins, or assist in their elimination via the chaperone-mediated autophagy or the ubiquitin proteasome system. HSPs have recently been classified into seven families, named HSPH (HSP110), HSPC (HSP90), HSPA (HSP70), HSPD/E (HSP60/HSP10), CCT (TRiC), DNAJ (HSP40) and HSPB (small HSP) [1,2]. Among them, HSP90 (HSPC) abundantly express in a variety type of unstressed cells and represents 1–2% of total cellular proteins, which increases to 4–6% under the stress conditions [2]. HSP90 consists of three domains, such as N-terminal domains, middle domains and C-terminal domains, and acts as an ATP-dependent chaperone [3]. It has been shown that HSP90 is overexpressed in many types of cancers, and that HSP90-dependent client proteins are involved in a variety of oncogenic pathways [4,5]. Therefore, inhibition of HSP90 functions has become as one of the leading strategies for anticancer chemotherapeutics [4,5]. In our previous study [6], we have demonstrated that HSP90 inhibitors such as geldanamycin [7], 17-allylamino-17demethoxy-geldanamycin (17-AAG) [8] and 17-dimethylamino-ethylamino-17-demethoxy-geldanamycin (17-DMAG) [9], cause epidermal growth factor receptor (EGFR) desensitization in human pancreatic cancer cells, and that the activation of p38 mitogen-activated protein (MAP) kinase induced by HSP90 inhibitors regulates the desensitization of EGFR via its phosphorylation at Ser1046/7. HSP90 inhibitors, by interfering the N-terminal domain ATP binding site of HSP90, cause the destabilization and eventual degradation of HSP90 client proteins, and then lead to inhibit ATP-dependent HSP90 chaperone activity [10]. Regarding the MAP kinase superfamily, it is generally recognized that p44/p42 MAP kinase, p38 MAP kinase and stress-activated protein kinase/c-*Jun* N-terminal kinase play central roles in a variety of cellular functions, including proliferation, differentiation and survival [11]. Therefore, HSP90 is considered to act as a pivotal modulator of various cellular functions via MAP kinases such as p38 MAP kinase.

Bone metabolism is strictly regulated by two types of antagonistic functional cells; osteoblasts and osteoclasts [12]. Bone tissue is continuously regenerated through a process so called bone remodeling [13]. To maintain an adequate bone quality and the quantity, osteoblastic bone formation and osteoclastic bone resorption are tightly coordinated. The disruption of bone remodeling process causes metabolic bone diseases such as osteoporosis or fracture healing distress. With regard to HSP90 inhibitor-effects on bone metabolism, 17-AAG reportedly stimulates osteoclast formation and promotes osteolytic bone metastasis in bone metastasis of a breast cancer cell line [14]. In addition, it has been shown that geldanamycin induces autophagy and apoptosis of osteosarcoma cells [15]. However, the exact roles of HSP90 in bone metabolism have not yet been fully clarified.

Interleukin-6 (IL-6) is a multifunctional cytokine which belongs to the glycoprotein 130 (gp130) cytokine family, and has important physiological effects on a variety of cell functions, such as the promotion of B-cell differentiation, the T-cell activation and the induction of acute-phase proteins [16,17]. It has been recognized that IL-6 stimulates bone resorption and induces osteoclast formation [17], and IL-6 reportedly plays a pivotal role in the process of bone fracture repair [18]. Thus, accumulating evidence suggests that IL-6 is an osteotropic modulator, and influence bone formation under the condition of increased bone turnover [19]. On the other hand, prostaglandins (PGs) modulate various bone cell functions as autacoids. Among them, PGF_2α_, which has been conventionally recognized as a potent bone-resorptive agent [20], is currently recognized as a bone remodeling mediator [21]. It has been previously reported that PGF_2α_ induced IL-6 production in osteoblast-enriched cultured neonatal mouse calvaria, resulting in bone resorption [20,22]. We have previously shown that PGF_2α_ stimulates the synthesis of IL-6 through p44/p42 MAP kinase and p38 MAP kinase in osteoblast-like MC3T3-E1 cells [23,24]. Thus, it is probable that the PGF_2α_-induced IL-6 synthesis is not specific for osteoblast-like MC3T3-E1 cells but general phenomena in osteoblasts. However, there is no report showing the roles of MAP kinases in the PGF_2α_-stimulated IL-6 synthesis in osteoblasts as far as we know. In addition, we have reported that Rho kinase inhibitors significantly suppress the synthesis of IL-6 and the phosphorylation of p38 MAP kinase induced by PGF_2α_ without affecting the levels of total p38 MAP kinase in these cells, suggesting that Rho-kinase plays a role in PGF_2α_-stimulated IL-6 synthesis as an upstream regulator of p38 MAP kinase [25]. These findings lead us to speculate that HSP90 could regulate the IL-6 synthesis stimulated by PGF_2α_ in osteoblasts.

In the present study, we investigated whether HSP90 is involved in the PGF_2α_-induced IL-6 synthesis in osteoblast-like MC3T3-E1 cells. We herein show that HSP90 inhibitors enhance the PGF_2α_-stimulated IL-6 synthesis in these cells, and that the amplifying effect is exerted through up-regulating p38 MAP kinase activation.

## Materials and methods

### Materials

Geldanamycin and PGF_2α_ were purchased from Sigma-Aldrich Co. (St. Louis, MO, USA). 17-AAG, 17-DMAG and SB203580 were obtained from Calbiochem-Novabiochem Co. (La Jolla, CA). Onalespib was purchased from Selleckchem (Houston, TX). Mouse interleukin-6 enzyme-linked immunosorbent assay (ELISA) kit was purchased from R&D System, Inc. (Minneapolis, MN). Phospho-specific p44/p42 MAP kinase antibodies, p44/p42 MAP kinase antibodies, phospho-specific p38 MAP kinase antibodies, p38 MAP kinase antibodies, phospho-specific myosin phosphatase targeting subunit (MYPT-1) antibodies, MYPT-1 antibodies, RhoA antibodies and Rho-kinase (ROCK1) antibodies were obtained from Cell Signaling Technology, Inc. (Beverly, MA). HSP90 antibodies and Glyceraldehyde-3-phosphate dehydrogenase (GAPDH) antibodies were purchased from Santa Cruz Biotechnology, Inc. (Santa Cruz, CA). An ECL Western blotting detection system was obtained from GE Healthcare Life Sciences (Chalfont, UK). Control short interfering RNA (siRNA; Silencer Negative Control no.1 siRNA) and HSP90-siRNA (Silencer select Pre-designed siRNA, s67897 and s67898, presented as #1 and #2) were purchased from Ambion (Austin, TX). Other materials and chemicals were obtained from commercial sources. PGF_2α_ was dissolved in ethanol. Geldanamycin, 17-AAG, 17-DMAG, onalespib and SB203580 were dissolved in dimethyl sulfoxide. The maximum concentration of ethanol or dimethyl sulfoxide was 0.1%, which did not affect the assay for IL-6, real-time RT-PCR or Western blot analysis.

### Cell culture

Cloned osteoblast-like MC3T3-E1 cells, an immortalized cell line which had been derived from newborn mouse calvaria [26] were maintained as previously described [27]. Briefly, the cells were cultured in α-minimum essential medium (α-MEM) containing 10% fetal bovine serum (FBS) at 37°C in a humidified atmosphere of 5% CO_2_/95% air. The cells in early passage until 21 passages were seeded into 35-mm diameter dishes (5 x 10^4^ cells/dish) or 90-mm diameter dishes (2 x 10^5^ cells/dish) in α-MEM containing 10% FBS. After 5 days, the medium was exchanged for α-MEM containing 0.3% FBS. The cells were used for experiments after 48 h.

### siRNA transfection

To knock down HSP90 in osteoblast-like MC3T3-E1 cells, the cells were transfected with HSP90-siRNA or negative control siRNA utilizing siLentFect according to the manufacturer’s protocol. In brief, the cells (2 x 10^5^ cells) were seeded into 90-mm diameter dishes in α-MEM containing 10% FBS, and sub-cultured for 48 h. The cells were then incubated at 37°C with 10 nM siRNA-siLentFect complexes (#1) or 30 nM siRNA-siLentFect complexes (#2). After 24 h, the medium was exchanged for α-MEM containing 0.3% FBS. The transfected cells were then stimulated by 10 μM of PGF_2α_ or vehicle in α-MEM containing 0.3% FBS for the indicated periods.

### Assay for IL-6

The cultured cells were stimulated by 10 μM of PGF_2α_ or vehicle in 1 ml of α-MEM containing 0.3% FBS for the indicated periods. When indicated, cells were pretreated with various doses of geldanamycin, 17-AAG, 17-DMAG or onalespib for 60 min. The preincubation with 30 μM of SB203580 or vehicle was performed for 60 min prior to the pretreatment. The cells were stimulated by 10 μM of PGF_2α_ or vehicle in 1 ml of α-MEM containing 0.3% FBS for the indicated periods. The conditioned medium was collected at the end of incubation, and the IL-6 concentration in the medium was then measured using the mouse IL-6 ELISA kit according to the manufacturer’s protocol.

### Reverse transcription-quantitative polymerase chain reaction (RT-qPCR)

The cultured cells were pretreated with 0.3 μM of geldanamycin, onalespib or vehicle for 60 min, and then stimulated by 10 μM of PGF_2α_ or vehicle in α-MEM containing 0.3% FBS for 3 h. Total RNA was isolated and reverse transcribed into complementary DNA using TRIzol reagent (Invitrogen; Thermo Fisher Scientific, Inc., Heysham, Lancashire, UK) and Omniscript Reverse Transcriptase kit (Qiagen Inc., Valencia, CA), respectively. RT-qPCR was performed in capillaries using a LightCycler system with the LightCycler FastStart DNA Master SYBR Green I (Roche Diagnostics, Basel, Switzerland). Sense and antisense primers for mouse IL-6 mRNA and GAPDH mRNA were purchased from Takara Bio Inc. (Tokyo, Japan) (primer set ID: MA039013 or RA015380, respectively). The amplified products were determined by melting curve analysis and agarose electrophoresis. The IL-6 mRNA levels were normalized to those of GAPDH mRNA.

### Western blot analysis

The cultured cells were pretreated with various doses of geldanamycin, 17-AAG or onalespib for 60 min, and then stimulated by 10 μM of PGF_2α_ or vehicle in α-MEM containing 0.3% FBS for the indicated periods. As for the HSP90 knockdown cells, the cells transfected with siRNA were stimulated by 10 μM of PGF_2α_ or vehicle in 1 ml of α-MEM containing 0.3% FBS for the indicated periods. The cells were then washed twice with phosphate-buffered saline, and then lysed, homogenized and sonicated in a lysis buffer containing 62.5 mM Tris/HCl, pH 6.8, 2% sodium dodecyl sulfate (SDS), 50 mM dithiothreitol and 10% glycerol. SDS-polyacrylamide gel electrophoresis (PAGE) was performed by the method of Laemmli [28] in 10% polyacrylamide gels. The protein was fractionated and transferred onto an Immun-Blot PVDF membrane (Bio-Rad, Hercules, CA). The membranes were blocked with 5% fat-free dry milk in Tris-buffered saline-Tween (TBS-T; 20 mM Tris-HCl, pH 7.6, 137 mM NaCl, 0.1% Tween 20) for 1 h before incubation with primary antibodies. Western blot analysis was performed as described previously [29] using phospho-specific p44/p42 MAP kinase antibodies, p44/p42 MAP kinase antibodies, phospho-specific p38 MAP kinase antibodies, p38 MAP kinase antibodies, HSP90 antibodies, phospho-specific MYPT-1 antibodies, MYPT-1 antibodies, RhoA antibodies, ROCK1 antibodies and GAPDH antibodies as primary antibodies with peroxidase-labeled antibodies raised in goat against rabbit IgG (KPL, Inc., Gaithersburg, MD) used as secondary antibodies. The primary and secondary antibodies were diluted at 1:1000 with 5% fat-free dry milk in TBS-T. The peroxidase activity on the PVDF sheet was visualized on X-ray film by means of the ECL Western blotting detection system.

### Densitometric analysis

A densitometric analysis of the Western blots was performed using a scanner and image analysis software program (image J version 1.48, National Institutes of Health, Bethesda, MD). The phosphorylated protein levels were calculated as follows: the background-subtracted signal intensity of each phosphorylation signal was respectively normalized to the total protein signal and plotted as the fold increase in comparison to that of the control cells without stimulation.

### Statistical analysis

The data were analyzed by ANOVA followed by Bonferroni method for multiple comparisons between pairs, and *p*<0.05 was considered to be statistically significant. All data are presented as the mean ± S.E.M. of triplicate determinations from three independent cell preparations.

## Results

### Effects of HSP90 inhibitors on the PGF_2α_-stimulated IL-6 release in MC3T3-E1 cells

**Fig 1:**
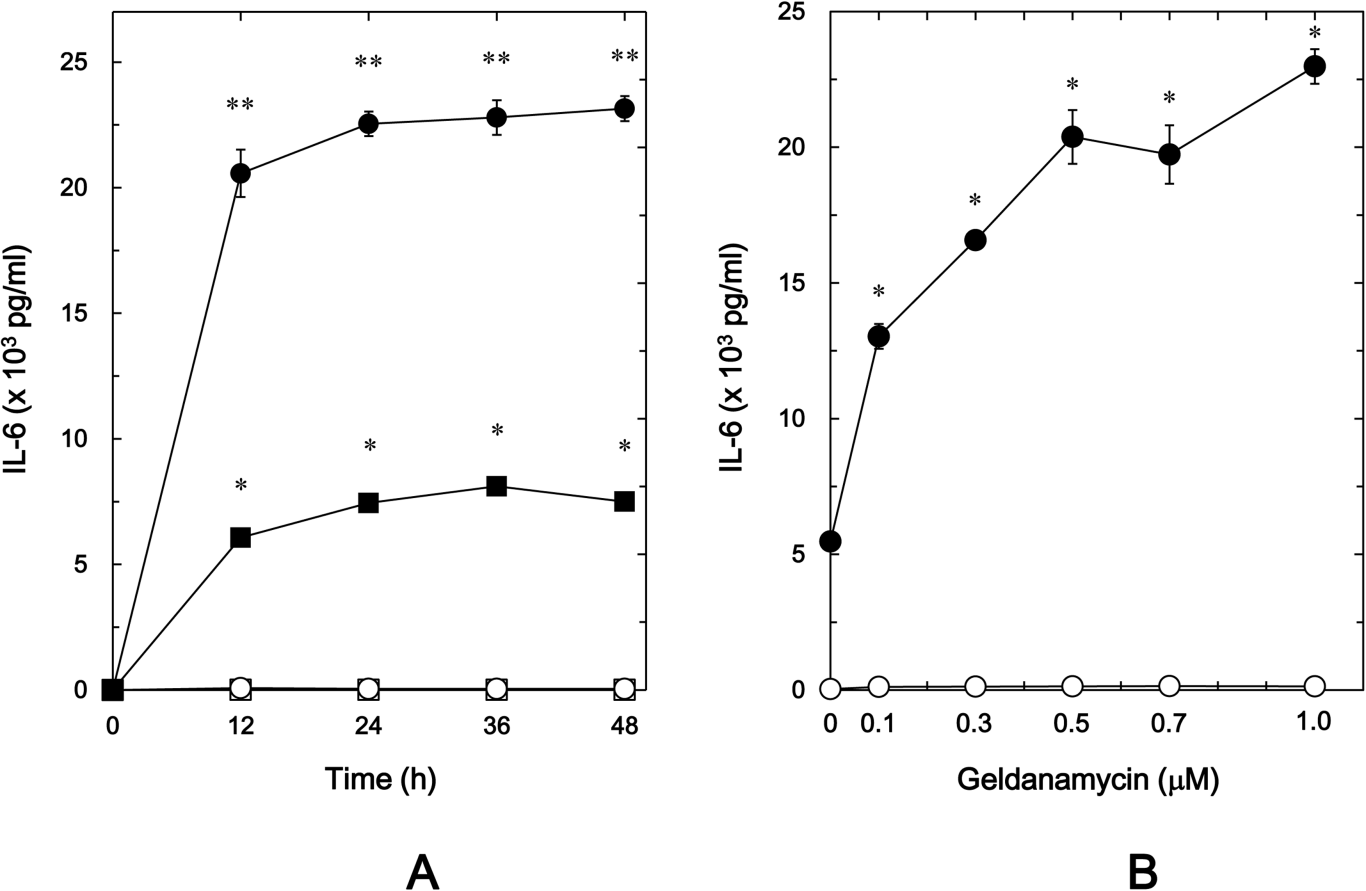
Effect of geldanamycin on the PGF_2α_-stimulated IL-6 release in MC3T3-E1 cells. (A) The cultured cells were pretreated with 1 μM of geldanamycin (●,○) or vehicle (■,□) for 60 min, and then stimulated by 10 μM of PGF_2α_ (●,■) or vehicle (○,□) for the indicated periods. (B) The cultured cells were pretreated with various doses of geldanamycin for 60 min, and then stimulated by 10 μM of PGF_2α_ (●) or vehicle (○) for 48 h. IL-6 concentrations in the conditioned medium were determined by ELISA. Each value represents the mean ± S.E.M. of triplicate determinations from three independent cell preparations. (A) ^*^p<0.05 compared to the value of control. ^**^p<0.05 compared to the value of PGF_2α_ alone. (B) ^*^p<0.05 compared to the value of PGF_2α_ alone.

**Fig 2:**
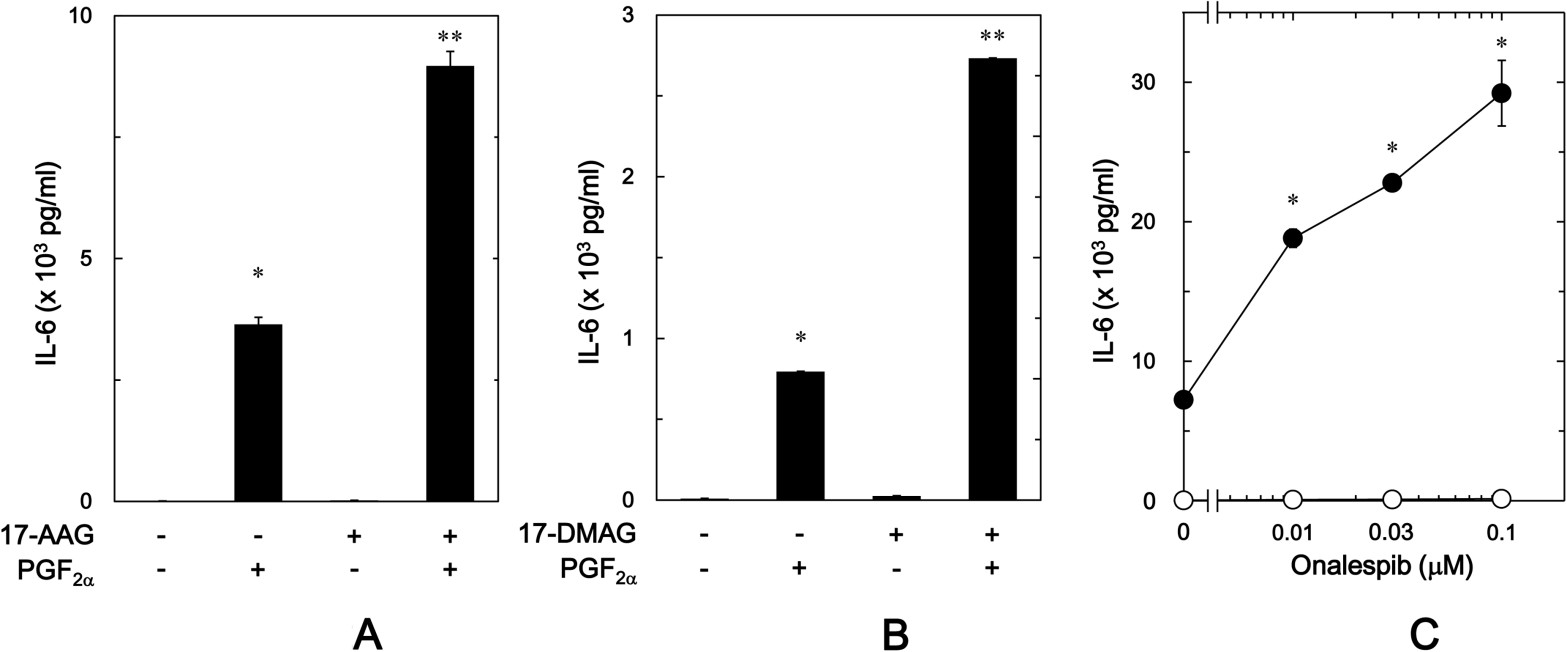
Effects of 17-AAG, 17-DMAG or onalespib on the PGF_2α_-stimulated IL-6 release in MC3T3-E1 cells. The cultured cells were pretreated with 1 μM of 17-AAG (A), 1 μM of 17-DMAG (B) or vehicle for 60 min, and then stimulated by 10 μM of PGF_2α_ or vehicle for 48 h. (C) The cultured cells were pretreated with various doses of onalespib for 60 min, and then stimulated by 10 μM of PGF_2α_ (●) or vehicle (○) for 48 h. IL-6 concentrations in the conditioned medium were determined by ELISA. Each value represents the mean ± S.E.M. of triplicate determinations from three independent cell preparations. (A,B) ^*^p<0.05 compared to the value of control. (A,B) ^**^p<0.05 compared to the value of PGF_2α_ alone. (C) ^*^p<0.05 compared to the value of PGF_2α_ alone.

It is recognized that geldanamycin, a benzoquinone ansamycin antibiotic, binds to the N-terminal domain ATP binding site of HSP90, inhibiting ATP-dependent HSP90 chaperone activity [7,10], and its less toxic derivative 17-AAG and 17-DMAG also bind specifically to HSP90 in a manner similar to geldanamycin [8,9]. In order to investigate the involvement of HSP90 in the PGF_2α_-induced synthesis of IL-6 in osteoblast-like MC3T3-E1 cells, we first examined the effects of these HSP90 inhibitors on the PGF_2α_-stimulated IL-6 release. Geldanamycin significantly amplified the PGF_2α_-stimulated IL-6 release in a time-dependent manner up to 48 h (Fig 1A). In addition, the enhancing effect of geldanamycin was dose-dependent in the range between 0.1 and 1 μM (Fig 1B). The maximum effect of geldanamycin was observed at 1 μM, which caused an approximately 330% increase in the PGF_2α_-effect. Additionally, 17-AAG significantly enhanced the PGF_2α_-stimulated IL-6 release (Fig 2A). The effect of 17-AAG (0.1 μM) on the IL-6 release caused an approximately 150% increase in the PGF_2α_-effect. Furthermore, 17-DMAG significantly amplified the IL-6 release (Fig 2B). The amplifying effect of 17-DMAG (0.1 μM) caused an approximately 240% increase in the PGF_2α_-effect.

It is known that 17-AAG and 17-DMAG belong to geldanamycin analogues [30], and onalespib is another type HSP90 inhibitor different from geldanamycin analogues, which binds to the N-terminal domain ATP binding site of HSP90 [31]. We further examined the effect of onalespib on the PGF_2α_-induced IL-6 release in osteoblast-like MC3T3-E1 cells. Onalespib, which by itself had little effect on the IL-6 release, significantly amplified the PGF_2α_-stimulated IL-6 release as well as geldanamycin analogues (Fig 2C). The enhancing effect of onalespib was dose-dependent in the range between 0.01 and 0.1 μM. Onalespib at 0.1 μM caused an approximately 300% increase in the PGF_2α_-effect.

### Effects of geldanamycin or onalespib on the PGF_2α_-induced expression of IL-6 mRNA in MC3T3-E1 cells

**Fig 3:**
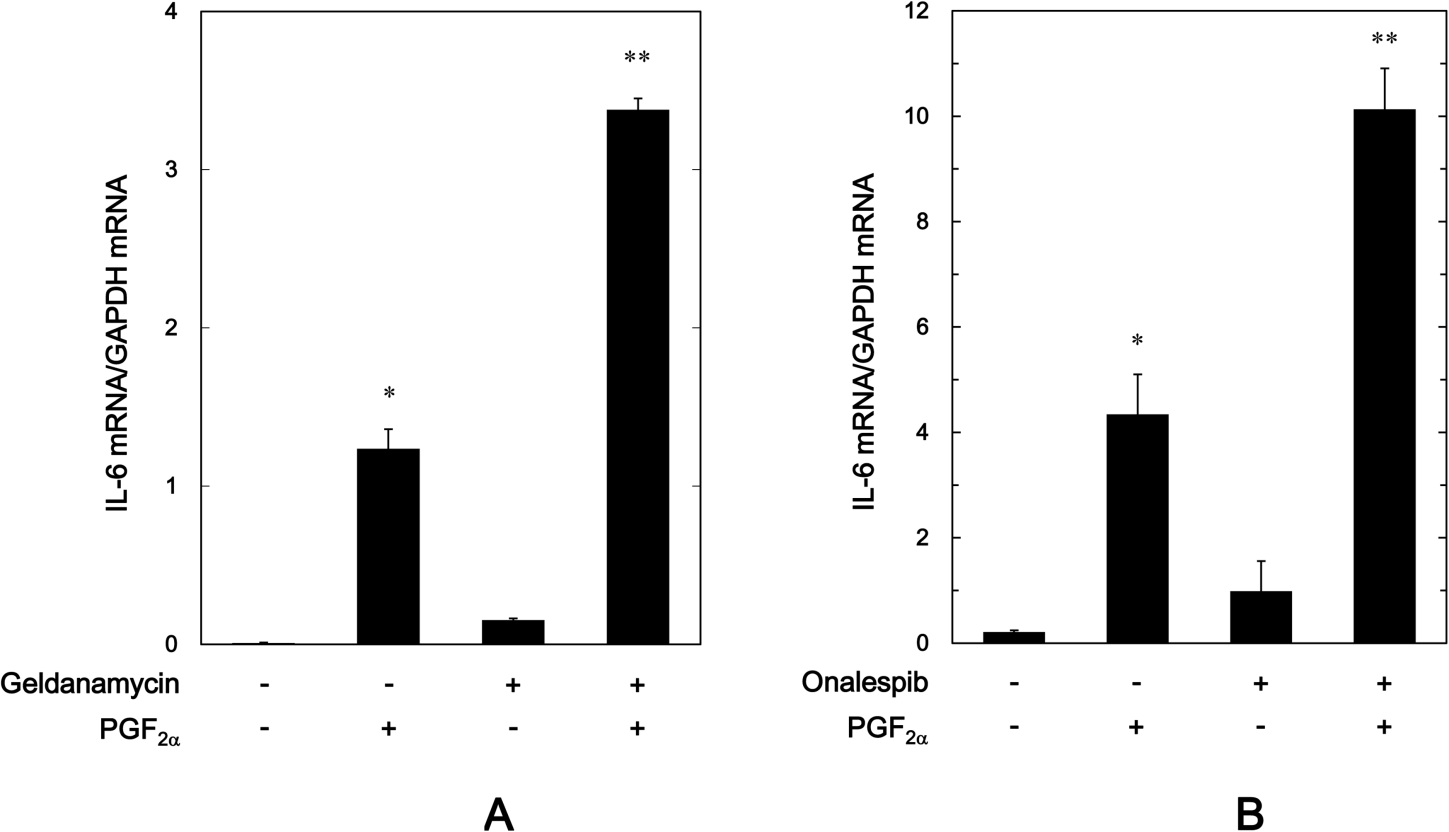
Effects of geldanamycin or onalespib on the PGF_2α_-induced expression levels of IL-6 mRNA in MC3T3-E1 cells. The cultured cells were pretreated with 0.3 μM of geldanamycin (A), onalespib (B) or vehicle for 60 min, and then stimulated by 10 μM of PGF_2α_ or vehicle for 3 h. The respective total RNA was then isolated and transcribed into cDNA. The expressions of IL-6 mRNA and GAPDH mRNA were quantified by RT-qPCR. The IL-6 mRNA levels were normalized to those of GAPDH mRNA. Each value represents the mean ± S.E.M. of triplicate determinations from three independent cell preparations. ^*^p<0.05 compared to the value of control. ^**^p<0.05 compared to the value of PGF_2α_ alone.

In order to clarify whether the amplifying effects of geldanamycin or onalespib on the PGF_2α_-stimulated IL-6 release is mediated through transcriptional events, we next examined the effects of geldanamycin or onalespib on the PGF_2α_-induced IL-6 mRNA expression. We found that geldanamycin and onalespib, which by themselves had little effect on the mRNA levels of IL-6, significantly up-regulated the expression levels of mRNA induced by PGF_2α_ (Fig 3A and Fig 3B).

### Effects of HSP90 inhibitors on the PGF_2α_-induced phosphorylation of p44/p42 MAP kinase in MC3T3-E1 cells

**Fig 4:**
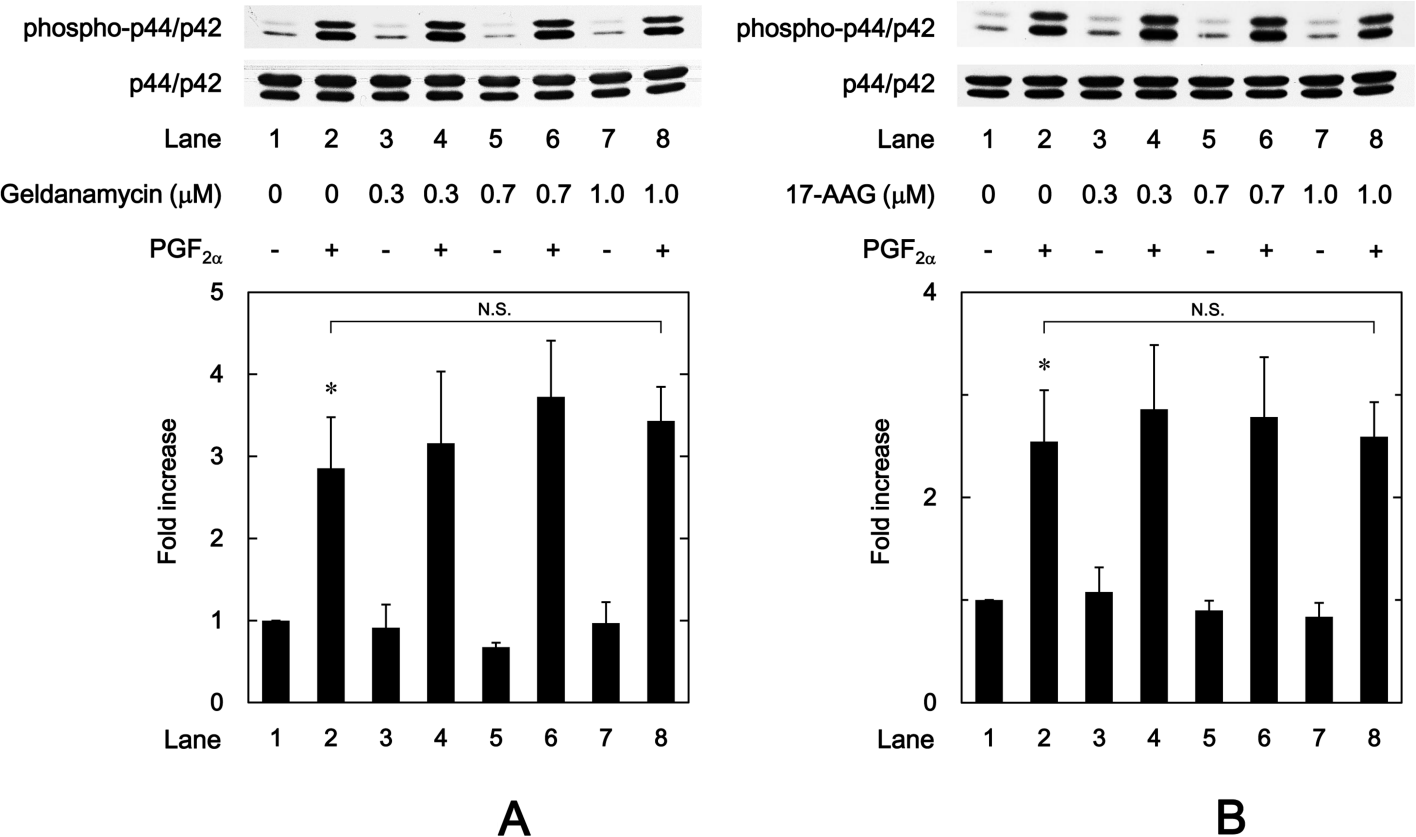
Effects of geldanamycin or 17-AAG on the PGF_2α_-induced phosphorylation of p44/p42 MAP kinase in MC3T3-E1 cells. The cultured cells were pretreated with various doses of geldanamycin (A) or 17-AAG (B) for 60 min, and then stimulated by 10 μM of PGF_2α_ or vehicle for 20 min. The cell extracts were then subjected to SDS-PAGE with subsequent Western blot analysis with antibodies against phospho-specific p44/p42 MAP kinase or p44/p42 MAP kinase. The histogram shows the quantitative representations of the levels of phosphorylated p44/p42 MAP kinase after normalization with respect to p44/p42 MAP kinase obtained from laser densitometric analysis. The levels were expressed as the fold increase to the basal levels presented as lane 1. Each value represents the mean ± S.E.M. of triplicate determinations from three independent cell preparations. ^*^p<0.05 compared to the value of control. N.S. designates no significant difference between the indicated pairs.

Regarding the intracellular signaling pathway of PGF_2α_ in osteoblasts, we have shown that p44/p42 MAP kinase acts as a positive regulator in the PGF_2α_-stimulated IL-6 synthesis in osteoblast-like MC3T3-E1 cells [23,24]. Therefore, we examined the effects of HSP90 inhibitors on the PGF_2α_-induced phosphorylation of p44/p42 MAP kinase in these cells. However, geldanamycin failed to affect the PGF_2α_-induced phosphorylation of p44/p42 MAP kinase (Fig 4A). In addition, 17-AAG had no effect on the PGF_2α_-induced phosphorylation of p44/p42 MAP kinase (Fig 4B).

### Effects of HSP90 inhibitors on the phosphorylation of p38 MAP kinase induced by PGF_2α_ in MC3T3-E1 cells

**Fig 5:**
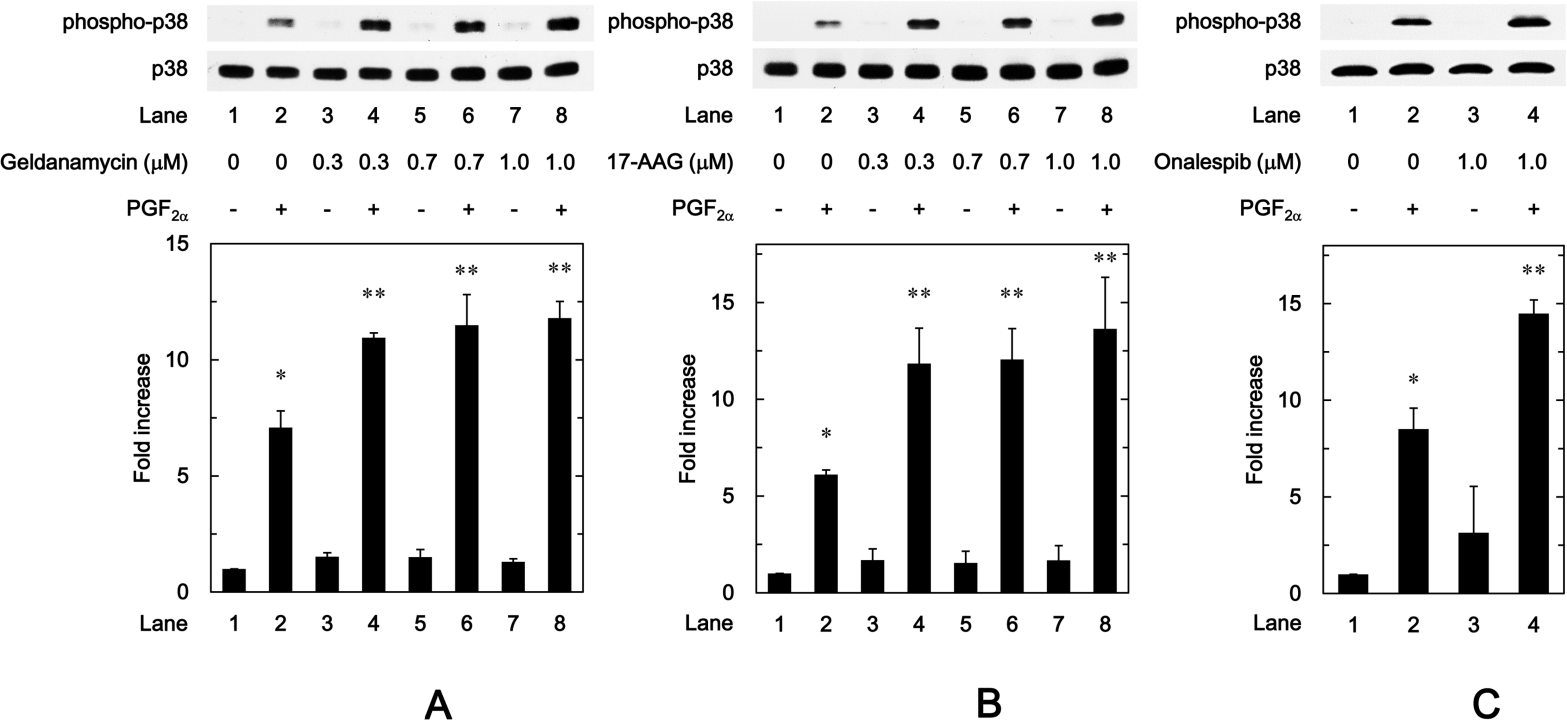
Effects of geldanamycin, 17-AAG or onalespib on the phosphorylation of p38 MAP kinase induced by PGF_2α_ in MC3T3-E1 cells. The cultured cells were pretreated with the indicated doses of geldanamycin (A) or 17-AAG (B) or onalespib (C) for 60 min, and then stimulated by 10 μM of PGF_2α_ or vehicle for 10 min. The cell extracts were then subjected to SDS-PAGE with subsequent Western blot analysis with antibodies against phospho-specific p38 MAP kinase or p38 MAP kinase. The histogram shows the quantitative representations of the levels of phosphorylated p38 MAP kinase after normalization with respect to p38 MAP kinase obtained from laser densitometric analysis. The levels were expressed as the fold increase to the basal levels presented as lane 1. Each value represents the mean ± S.E.M. of triplicate determinations from three independent cell preparations. ^*^p<0.05 compared to the value of control. ^**^p<0.05 compared to the value of PGF_2α_ alone.

In our previous study [24], we demonstrated that PGF_2α_ stimulates IL-6 synthesis at least in part through p38 MAP kinase in addition to p44/p42 MAP kinase in osteoblast-like MC3T3-E1 cells. In order to clarify whether the activation of p38 MAP kinase is implicated in the enhancement by HSP90 inhibitors of the PGF_2α_-induced IL-6 synthesis in MC3T3-E1 cells, we examined the effects of HSP90 inhibitors on the PGF_2α_-induced phosphorylation of p38 MAP kinase. Geldanamycin, which alone had little effect on the p38 MAP kinase phosphorylation, significantly strengthened the PGF_2α_-induced phosphorylation of p38 MAP kinase in a dose-dependent manner in the range between 0.3 and 1.0 μM (Fig 5A). Additionally, 17-AAG as well as geldanamycin, which by itself did not affect the p38 MAP kinase phosphorylation, markedly enhanced the PGF_2α_-induced phosphorylation of p38 MAP kinase (Fig 5B). The amplifying effect of 17-AAG on the p38 MAP kinase phosphorylation was dose-dependent in the range between 0.3 and 1.0 μM. Furthermore, we found that onalespib (1 μM), as well as geldanamycin and 17-AAG, which by itself had little effect of the p38 MAP kinase phosphorylation, markedly enhanced the PGF_2α_-induced phosphorylation of p38 MAP kinase (Fig 5C).

### Effects of HSP90 inhibitors on the PGF_2α_-induced phosphorylation of MYPT-1, and the protein levels of MYPT-1, RhoA and Rho-kinase in MC3T3-E1 cells

**Fig 6:**
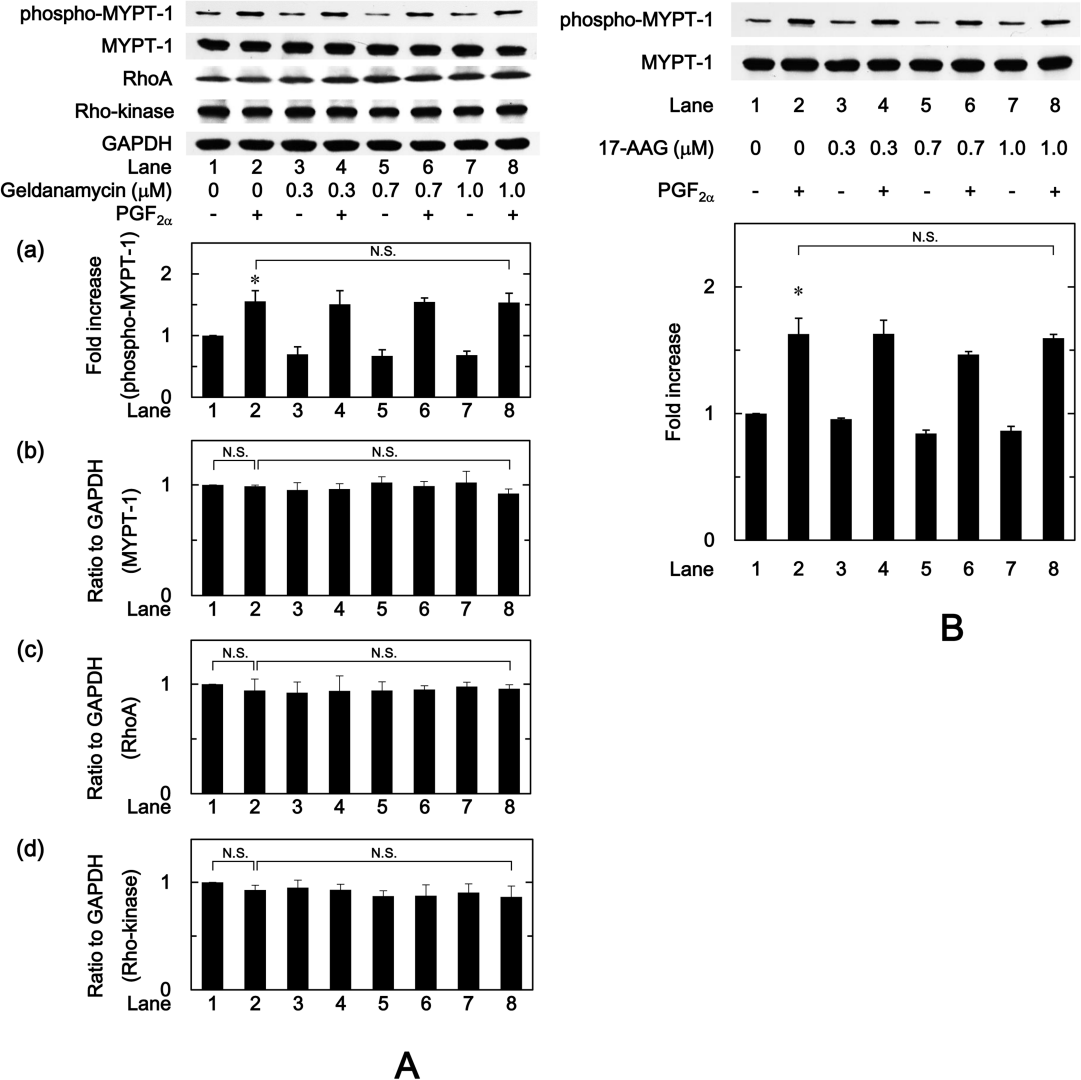
Effects of geldanamycin or 17-AAG on the phosphorylation of MYPT-1, the amounts of MYPT-1, RhoA and Rho-kinase induced by PGF_2α_ in MC3T3-E1 cells. The cultured cells were pretreated with various doses of geldanamycin (A) or 17-AAG (B) for 60 min, and then stimulated by 10 μM of PGF_2α_ or vehicle for 2 min. The cell extracts were then subjected to SDS-PAGE with subsequent Western blot analysis with antibodies against phospho-specific MYPT-1, MYPT-1, RhoA or Rho-kinase. (a) The histogram shows the quantitative representations of the levels of phosphorylated MYPT-1 after normalization with respect to MYPT-1 obtained from laser densitometric analysis. The levels were expressed as the fold increase to the basal levels presented as lane 1. (b),(c),(d) The histogram shows the quantitative representations of the levels of MYPT-1 (b), RhoA (c) and Rho-kinase (d) after normalization with respect to GAPDH obtained from laser densitometric analysis, respectively. The levels were expressed as the ratio to the levels presented as lane 1. Each value represents the mean ± S.E.M. of triplicate determinations from three independent cell preparations. ^*^p<0.05 compared to the value of control. N.S. designates no significant difference between the indicated pairs.

In addition to our previous findings in the PGF_2α_-stimulated IL-6 synthesis in osteoblast-like MC3T3-E1 cells, we have demonstrated that Rho-kinase positively regulates at a point upstream of p38 MAP kinase [25]. It is generally recognized that MYPT-1, which is a component of myosin phosphatase, is a substrate of Rho-kinase [32,33]. We next examined the effects of HSP90 inhibitors on the PGF_2α_-induced phosphorylation of MYPT-1, and the protein levels of MYPT-1, RhoA and Rho-kinase in MC3T3-E1 cells. Geldanamycin failed to affect the PGF_2α_-induced phosphorylation of MYPT-1 (Fig 6A). We found that the protein levels of MYPT-1, RhoA or Rho-kinase were not affected by geldanamycin with or without PGF_2α_ stimulation (Fig 6A). 17-AAG, as well as geldanamycin, had no effect on the PGF_2α_-induced phosphorylation of MYPT-1 (Fig 6B). In addition, we confirmed that 17-AAG did not affect the amounts of MYPT-1, RhoA or Rho-kinase (data not shown).

### Effect of PGF_2α_ on the phosphorylation of p38 MAP kinase in HSP90 knockdown MC3T3-E1 cells

**Fig 7:**
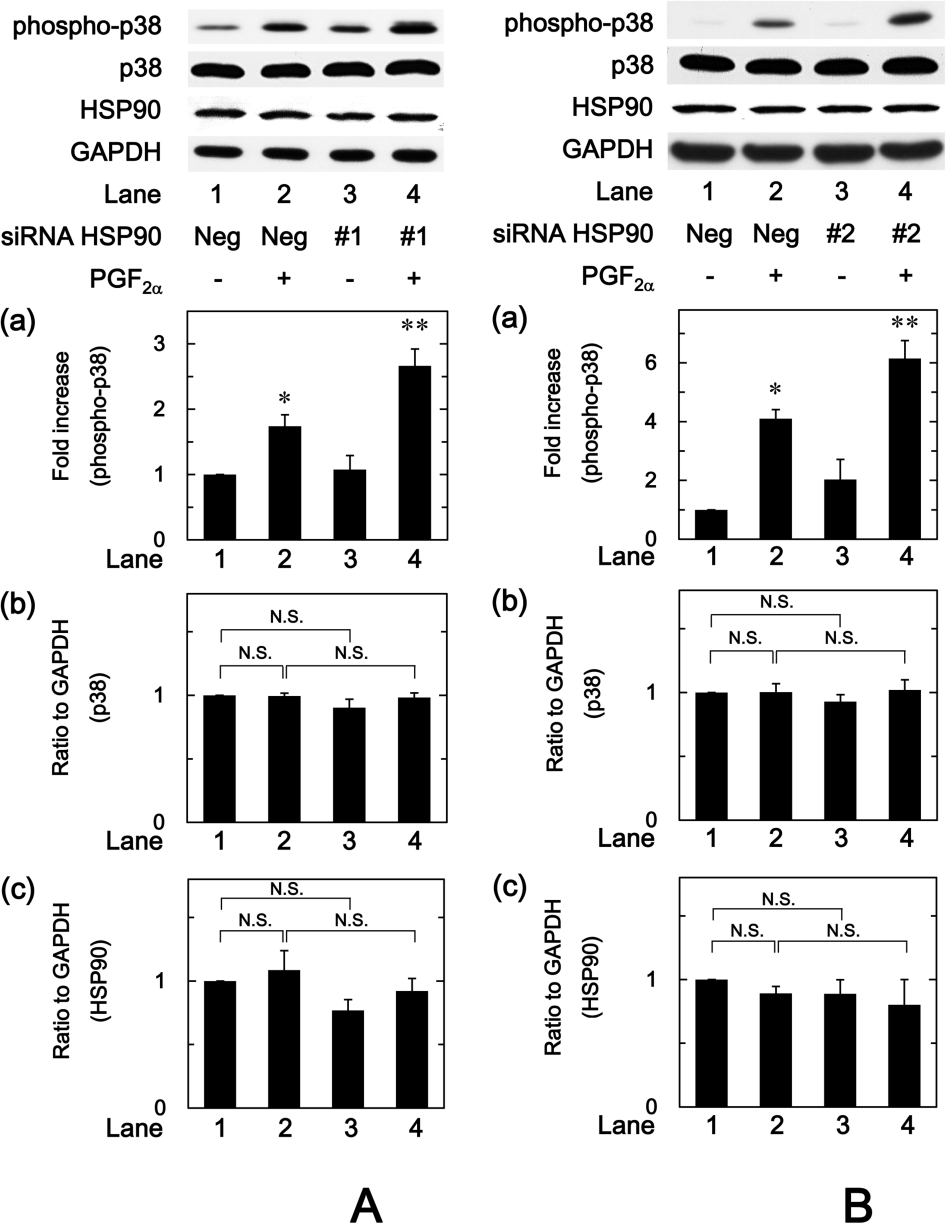
Effect of PGF_2α_ on the phosphorylation of p38 MAP kinase in HSP90 knockdown MC3T3-E1 cells. (A) The cultured cells were transfected with 10 nM negative control siRNA (Neg) or 10 nM HSP90-siRNA (#1). (B) The cultured cells were transfected with 30 nM negative control siRNA (Neg) or 30 nM HSP90-siRNA (#2). Twenty-four hours after transfection, the cells were stimulated by 10 μM PGF_2α_ or vehicle for 10 min. The cell extracts were then subjected to SDS-PAGE with subsequent Western blot analysis with antibodies against phospho-specific p38 MAP kinase, p38 MAP kinase, HSP90 or GAPDH. (a) The histogram shows the quantitative representations of the levels of phosphorylated p38 MAP kinase after normalization with respect to GAPDH obtained from laser densitometric analysis. The levels were expressed as the fold increase to the basal levels presented as lane 1. (b),(c) The histogram shows the quantitative representations of the levels of (b) total p38 MAP kinase and (c) HSP90αβ after normalization with respect to GAPDH obtained from laser densitometric analysis, respectively. The levels were expressed as the ratio to the levels presented as lane 1. Each value represents the mean ± S.E.M. of triplicate determinations from three independent cell preparations. ^*^p<0.05 compared to the value of control. ^**^p<0.05 compared to the value of PGF_2α_ alone.

To further investigate whether HSP90 affects the PGF_2α_-induced phosphorylation of p38 MAP kinase in osteoblast-like MC3T3-E1 cells, we examined the effect of HSP90-siRNA on the phosphorylation of p38 MAP kinase induced by PGF_2α_. We found that the levels of HSP90 were not significantly but slightly reduced in the HSP90-siRNA (#1 and #2)-transfected cells (Fig 7A and Fig 7B). The levels of phosphorylated p38 MAP kinase induced by PGF_2α_ in the HSP90-siRNA transfected cells (#1 and #2) were significantly amplified compared to those in the control cells (Fig 7A and Fig 7B). Thus, it seems likely that HSP90-siRNA has little effect on HSP90 protein levels but reduces HSP90 activity in MC3T3-E1 cells. The protein levels of p38 MAP kinase were not affected by HSP90-siRNA (#1 and #2).

### Effect of SB203580 on the enhancement by HSP90 inhibitors of the PGF_2α_-stimulated IL-6 release in MC3T3-E1 cells

**Fig 8:**
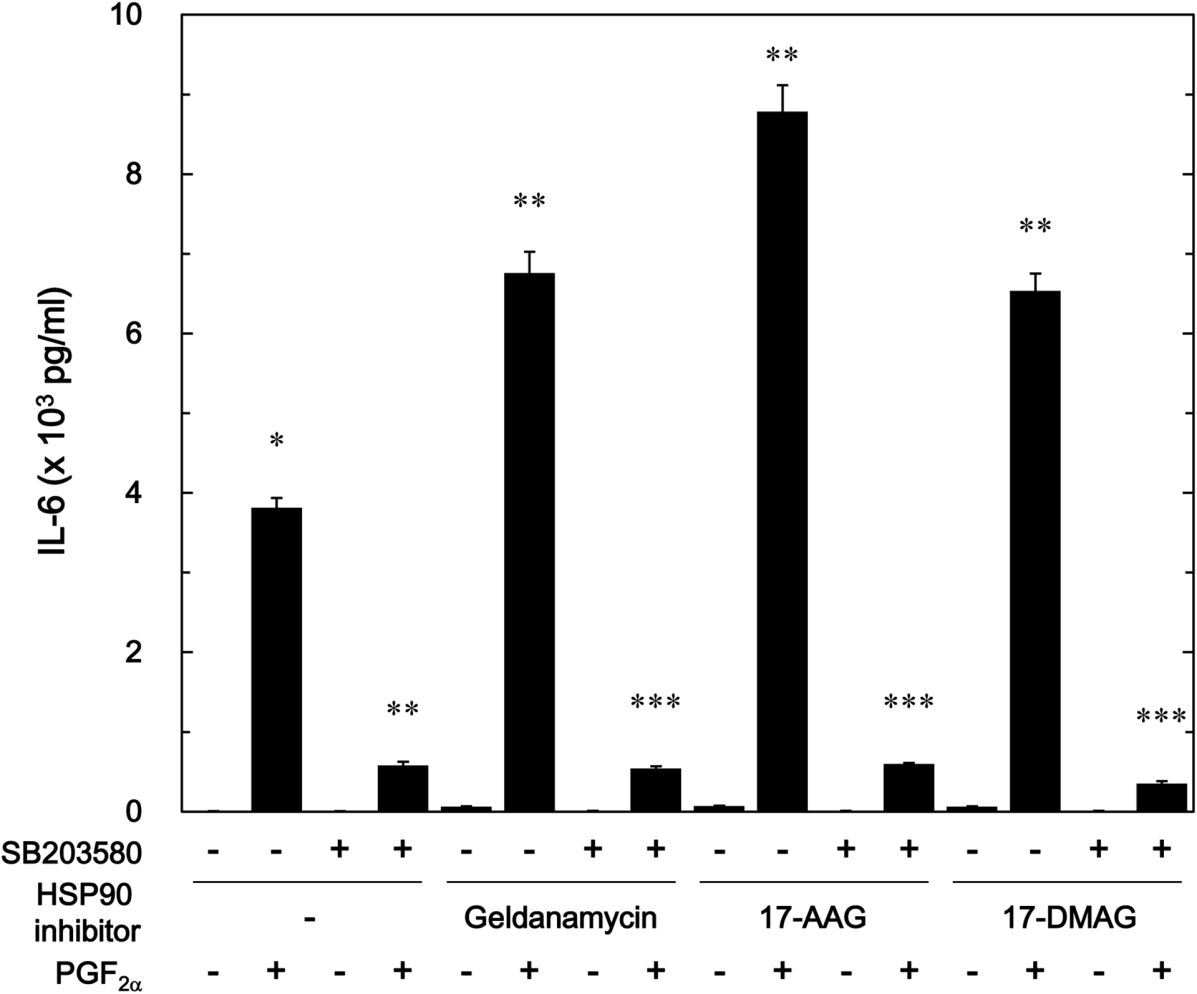
Effect of SB203580 on the enhancement by geldanamycin, 17-AAG or 17-DMAG of the PGF_2α_-induced IL-6 release in MC3T3-E1 cells. The cultured cells were preincubated with 30 μM of SB203580 or vehicle for 60 min, subsequently pretreated with 1 μM of geldanamycin, 1 μM of 17-AAG, 1 μM of 17-DMAG or vehicle for 60 min, and then stimulated by 10 μM of PGF_2α_ or vehicle for 48 h. IL-6 concentrations of the conditioned mediums were determined by ELISA. Each value represents the mean ± S.E.M. of triplicate determinations from three independent cell preparations. ^*^p<0.05 compared to the value of control. ^**^p<0.05 compared to the value of PGF_2α_ alone. ^***^p<0.05 compared to the value of PGF_2α_ with the pretreatment of each HSP90 inhibitor.

Furthermore, we examined the effect of SB203580, a p38 MAP kinase inhibitor [34], on the enhancement by HSP90 inhibitors of the PGF_2α_-stimulated IL-6 release in osteoblast-like MC3T3-E1 cells. We found that SB203580, which by itself had little effect on IL-6 levels, truly suppressed the PGF_2α_-induced IL-6 release as our previous report [25] (Fig 8). SB203580 markedly reduced the amplification by geldanamycin, 17-AAG or 17-DMAG of the PGF_2α_-stimulated IL-6 release (Fig 8). SB203580 (30 μM) caused an approximately 90%, 90% and 95% decrease in the effect of PGF_2α_ with geldanamycin, 17-AAG and 17-DMAG, respectively.

## Discussion

In the present study, we demonstrated that HSP90 inhibitors including geldanamycin, 17-AAG and 17-DMAG significantly enhanced the PGF_2α_-stimulated release of IL-6 in osteoblast-like MC3T3-E1 cells. We also found that onalespib, another type HSP90 inhibitor [31], amplified the IL-6 release induced by PGF_2α_ in these cells. In addition, we showed that the expression levels of IL-6 mRNA induced by PGF_2α_ were markedly amplified by geldanamycin and onalespib. Thus, it seems likely that the amplifying effect of HSP90 inhibitors on the PGF_2α_-stimulated IL-6 release is mediated through the gene transcription in MC3T3-E1 cells. Based on our findings, it is probable that HSP90 plays an inhibitory role in the PGF_2α_-stimulated IL-6 synthesis in osteoblast-like MC3T3-E1 cells. To the best of our knowledge, this is probable the first report showing the suppression by HSP90 of IL-6 synthesis in osteoblasts. Therefore, we next investigated the exact mechanism behind the suppression by HSP90 of the PGF_2α_-stimulated IL-6 synthesis in osteoblast-like MC3T3-E1 cells.

**Fig 9:**
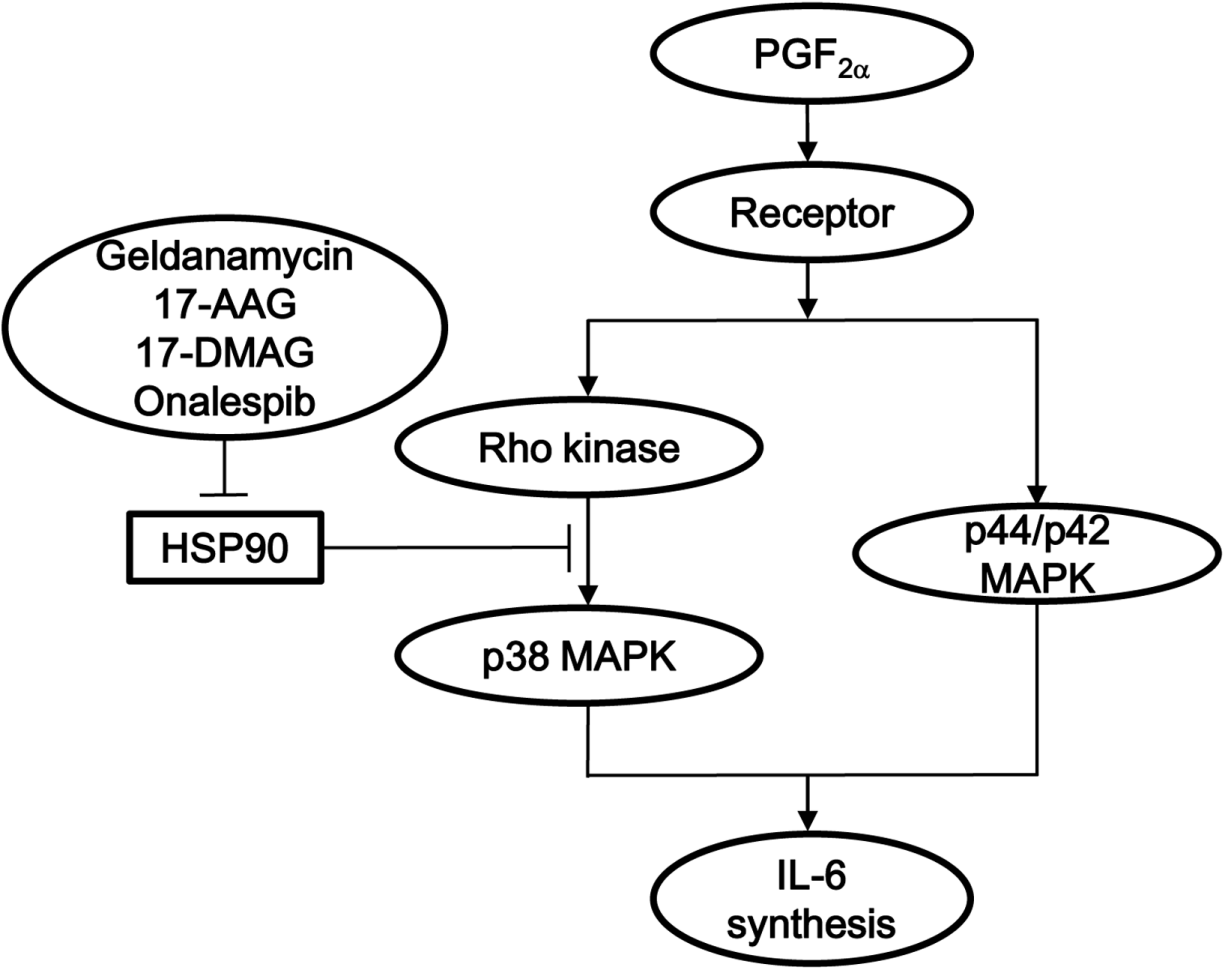
Schematic illustration of the regulatory mechanism underlying the amplifying effect of HSP90 inhibitors on the PGF_2α_-induced IL-6 synthesis in osteoblast-like MC3T3-E1 cells. PGF_2α_, prostaglandin F_2α_; MAPK, mitogen-activated protein kinase; HSP90, heat shock protein 90; IL-6, interleukin-6.

Regarding the intracellular signaling system of PGF_2α_ in osteoblasts, we have previously shown that PGF_2α_ stimulates the synthesis of IL-6 through p44/p42 MAP kinase and p38 MAP kinase in osteoblast-like MC3T3-E1 cells [23,24]. Additionally, in our previous study [25], we have reported that Rho-kinase positively regulates PGF_2α_-stimulated IL-6 synthesis at a point upstream of not p44/p42 MAP kinase but p38 MAP kinase in MC3T3-E1 cells. Thus, we investigated whether HSP90 inhibitors affect the PGF_2α_-stimulated activation of p44/p42 MAP kinase or p38 MAP kinase in these cells. However, neither geldanamycin nor 17-AAG affected the phosphorylation of p44/p42 MAP kinase. On the contrary, we demonstrated that geldanamycin, 17-AAG and onalespib significantly enhanced the PGF_2α_-stimulated phosphorylation of p38 MAP kinase. Therefore, it is probable that the enhancement by HSP90 inhibitors of PGF_2α_-stimulated IL-6 synthesis is due to up-regulating the activation of p38 MAP kinase but not p44/p42 MAP kinase in MC3T3-E1 cells. In addition, we demonstrated that either geldanamycin or 17-AAG failed to affect the PGF_2α_-induced phosphorylation of MYPT-1, a substrate of Rho-kinase [33]. We also found that HSP90 inhibitors had no effect of the amounts of MYPT-1, RhoA or Rho-kinase. It is generally recognized that Rho and the down-stream effector, Rho-kinase play crucial roles in a variety of cellular functions such as cell motility and smooth muscle contraction [32,33]. As for osteoblasts, it has been shown that the inhibition of RhoA-Rho-kinase signaling influences osteoblast adhesion, differentiation and mineralization [35]. In the present study, we showed that HSP90 inhibitors failed to affect the phosphorylation of MYPT-1, a target of Rho-kinase. Based on our findings, it seems unlikely that the amplification by HSP90 inhibitors of PGF_2α_-stimulated IL-6 synthesis is mediated through the enhancement of RhoA-Rho-kinase activity. Our findings suggest that HSP90 might function as a negative regulator in the PGF_2α_-stimulated IL-6 synthesis in osteoblast-like MC3T3-E1 cells, and that the effect of HSP90 on the IL-6 synthesis is exerted at the point between Rho-kinase and p38 MAP kinase. In order to further elucidate whether HSP90 regulates the PGF_2α_-induced activation of p38 MAP kinase in MC3T3-E1 cells, we examined the effect of HSP90-siRNA on the PGF_2α_-induced phosphorylation of p38 MAP kinase. The PGF_2α_-induced levels of phosphorylated p38 MAP kinase were significantly enhanced by HSP90-siRNA. Additionally, we clearly demonstrated that SB203580 reduced the amplification by geldanamycin, 17-AAG or 17-DMAG of the PGF_2α_-stimulated IL-6 release in MC3T3-E1 cells. Taking these findings into account, it is most likely that HSP90 limits the PGF_2α_-stimulated IL-6 synthesis in osteoblast-like MC3T3-E1 cells, and the suppressive effect of HSP90 is exerted at the point between Rho-kinase and p38 MAP kinase. The potential mechanism underlying amplification by HSP90 inhibitors of the PGF_2α_-stimulated IL-6 synthesis in osteoblasts is summarized as Fig 9.

In bone metabolism, it has been generally recognized that IL-6 acts as a potent bone resorptive agent and to promote osteoclast formation [17]. In addition to the IL-6-effect on bone resorption, IL-6 is currently considered as an osteotropic factor under the condition of increased bone turnover, and is inducible even bone formation [19]. Bone resorption is the primary step of bone remodeling, and bone formation is subsequently initiated [36]. To obtain the quantity of bone and the quality, the adequate handling of bone remodeling process performed by osteoclasts and osteoblasts is essential. Although there is no doubt that IL-6 stimulates osteoclastic bone resorption, IL-6 is recognized to act as a bone remodeling mediator from the viewpoint of whole bone metabolism. As for HSP90 in osteoblasts, we have shown that the expression levels of HSP90 protein are quite high in osteoblast-like MC3T3-E1 cells [37]. Therefore, our present findings, showing that HSP90 inhibitors could function as an up-regulator with regard to the PGF_2α_-stimulated IL-6 synthesis in osteoblasts, probably provides a novel insight with regard to HSP90 as an essential regulator of bone remodeling. Several HSP90 inhibitors including geldanamycin have been recognized as an anticancer drug, and the effects of HSP90 inhibitors have been tested in the clinical trial [38]. Based on our present findings, it is probable that HSP90 inhibitors, in addition to the anticancer chemotherapeutics, could affect bone metabolism as a bone remodeling modulator through the enhancement of IL-6 synthesis in osteoblasts. Therefore, HSP90 inhibitors might lead a new therapeutic strategy for acceleration of fracture healing and bone metabolic diseases such as osteoporosis. Further investigations would be required to clarify the details underlying the effects of HSP90 on bone metabolism.

In conclusion, our results strongly suggest that HSP90 negatively regulates the PGF_2α_-stimulated IL-6 synthesis in osteoblasts, and that the effect of HSP90 on the IL-6 synthesis is exerted through regulating p38 MAP kinase activation.
